# Breast Cancer Cells and PD-1/PD-L1 Blockade Upregulate the Expression of PD-1, CTLA-4, TIM-3 and LAG-3 Immune Checkpoints in CD4^+^ T Cells

**DOI:** 10.3390/vaccines7040149

**Published:** 2019-10-12

**Authors:** Reem Saleh, Salman M Toor, Sarah Khalaf, Eyad Elkord

**Affiliations:** 1 Cancer Research Center, Qatar Biomedical Research Institute (QBRI), Hamad Bin Khalifa University (HBKU), Qatar Foundation (QF), Doha 34110, Qatar; rsaleh@hbku.edu.qa (R.S.); mstoor@hbku.edu.qa (S.M.T.); 2 College of Health & Life Sciences, Hamad Bin Khalifa University, Qatar Foundation, Doha 34110, Qatar; skhalaf@mail.hbku.edu.qa; 3 Institute of Cancer Sciences, University of Manchester, Manchester M20 4GJ, UK

**Keywords:** breast cancer, anti-PD-1, anti-PD-L1, Tregs, immune checkpoints

## Abstract

Triple negative breast cancer (TNBC) is the most aggressive breast cancer subtype, and it exhibits resistance to common breast cancer therapies. Immune checkpoint inhibitors (ICIs) targeting programmed cell death 1 (PD-1) and its ligand, PD-L1, have been approved to treat various cancers. However, the therapeutic efficacy of targeting PD-1/PD-L1 axis in breast cancer is under clinical investigation. In addition, the mechanisms of action of drugs targeting PD-1 and PD-L1 have not been fully elucidated. In this study, we investigated the effect of human TNBC cell lines, MDA-MB-231 and MDA-MB-468, and the non-TNBC cell line, MCF-7, on the expression of immune checkpoints (ICs) on CD4^+^ T cell subsets, including regulatory T cells (Tregs), using a co-culture system. We also examined the effect of blocking PD-1 or PD-L1 separately and in combination on IC expression by CD4^+^ T cell subsets. We found that breast cancer cells upregulate the expression of ICs including PD-1, cytotoxic T lymphocyte-associated antigen-4 (CTLA-4), T cell immunoglobulin and mucin domain-containing protein 3 (TIM-3) and lymphocyte activation gene-3 (LAG-3) in CD4^+^ T cell subsets. We also found that the co-blockade of PD-1 and PD-L1 further upregulates the co-expression of TIM-3 and LAG-3 on CD4^+^CD25^+^ T cells and CD4^+^CD25^+^FoxP3^+^Helios^+^ Tregs in the presence of TNBC cells, but not in non-TNBC cells. Our results indicate the emergence of compensatory inhibitory mechanisms, most likely mediated by Tregs and activated non-Tregs, which could lead to the development of TNBC resistance against PD-1/PD-L1 blockade.

## 1. Introduction 

Breast cancer is one of the most common malignancies in females, associated with high morbidity rates all over the world [1]. Breast tumors are heterogeneous; they display a diverse range of histological grades, therapeutic responses and clinical outcomes depending on their molecular profiles and cell types [2,3]. Triple negative breast cancer (TNBC) is the most aggressive type of breast cancer; it exhibits high histopathological grades, high rates of distant metastasis, poor survival rates and accounts for 10–20% of all invasive breast cancer cases [4]. TNBC is characterized by the lack of expression of estrogen receptor (ER), human epidermal growth factor receptor-2 (HER2) and progesterone receptor (PR), hence it is resistant to conventional breast cancer therapies [4,5].

Immunotherapy in the form of immune checkpoint inhibitors (ICIs), such as monoclonal antibodies (mAbs) targeting PD-1 and PD-L1, have revolutionized the treatment for various cancer types [6,7,8,9,10]. PD-1, a co-inhibitory receptor, is expressed by activated T and B lymphocytes, natural killer (NK) cells and myeloid cells [11]. PD-1 has two ligands; PD-L1 which is primarily expressed on tumor cells, and can also be expressed on some subsets of myeloid cells and antigen-presenting cells (APCs), and PD-L2 which is predominantly expressed on APCs [11]. Inhibitory signals are generated upon PD-1/PD-L1 binding, which suppress the anti-tumor immune responses by inducing T cell apoptosis, and inhibiting cytotoxic T cell activation and cytokine production [12]. 

Most breast tumors are less immunogenic, characterized by low T cell infiltrates [13]. Amongst breast cancer subtypes, TNBC exhibits a high number of intratumoral and stromal tumor infiltrating lymphocytes (TILs), suggesting that immunotherapy could be beneficial for TNBC treatment [13]. PD-L1 expression has been detected in 20% of TNBC cases [13,14]. A positive correlation between PD-L1 expression and frequency of TILs, histological grades and poor survival rates has been observed in breast cancer patients [15,16,17]. Based on pre-clinical and clinical studies, it was suggested that PD-L1 could be a good prognostic marker and therapeutic target for breast cancer [17,18,19]. Clinical trials are currently underway to examine the therapeutic efficacy of targeting PD-1 and PD-L1 in breast cancer patients, including those with TNBC [13,20]. Recently, atezolizumab (anti-PD-L1 mAb) has been approved by the Federal Food and Drugs Administration (FDA) to be used for the treatment of PD-L1^+^ unresectable locally advanced or metastatic TNBC in combination with chemotherapy [21,22]. However, the therapeutic efficacy of atezolizumab as a “monotherapy”, and in other patient groups remains questionable [21]. Despite this, the effects of anti-PD-1 and anti-PD-L1 mAbs on the cellular and molecular components of the tumor microenvironment (TME) have not been fully elucidated. In particular, it is important to understand how these mAbs alter the immune phenotype of T cell subsets, such as Tregs, which are known for their central role in orchestrating immunosuppression within the TME, and promoting cancer progression [23].

In this study, we aimed to examine the effect of breast cancer cells on the expression level of ICs on CD4^+^ T cell subsets, including CD4^+^CD25^−^, CD4^+^CD25^+^, and CD4^+^CD25^+^FoxP3^+^Helios^+^ Tregs, in the absence and presence of anti-PD-1 mAb, anti-PD-L1 mAb or both mAbs. To do this, we utilized a co-culture system; activated PBMCs (peripheral blood mononuclear cells) from healthy donors were co-cultured with human TNBC cells (MDA-MB-231 and MDA-MB-468) or with non-TNBC cells (MCF-7). Each breast cancer cell line differentially express PD-L1; ~100% expression in MDA-MB-231, ~70% in MCF-7, and ~40% in MBA-MB-468 cells [24]. We determined the kinetics and level of expression of key inhibitory ICs (PD-1, CTLA-4, TIM-3 and LAG-3) and Treg-related markers (FoxP3 and Helios) in CD4^+^ T cells in the absence or presence of breast cancer cells.

## 2. Materials and Methods

### 2.1. Sample Collection and Isolation of Peripheral Blood Mononuclear Cells 

This study was performed under an ethical approval from Qatar Biomedical Research Institute, Doha, Qatar (Protocol no. 2017-006). All healthy donors provided written informed consent prior to sample collection, and all experiments were performed in accordance with relevant guidelines and regulations. Blood was collected from healthy donors and PBMCs were isolated by density gradient centrifugation using Histopaque-1077 (Sigma-Aldrich, Missouri, USA). PBMCs were frozen in cryovials at a density of 5 × 10^6^ cells in 1 mL of freezing medium [10% dimethylsulphoxide (DMSO; Sigma-Aldrich), 50% fetal calf serum (FCS; Hyclone, GE Healthcare Life Sciences, Utah, USA) and 40% RPMI-1640 medium (Life Technologies, New York, NY, USA)], and stored in liquid nitrogen to be used in batches in subsequent experiments. 

### 2.2. Cell Lines

Human breast cancer cell lines, MDA-MB-231, MDA-MB-468 and MCF-7, from American Type culture collection (ATCC, Maryland, USA), were authenticated by STR Fingerprinting at the Regional Facility for DNA Fingerprinting, Rajiv Gandhi Centre for Biotechnology, India. Cells were maintained and cultured in complete RPMI-1640 medium supplemented with 10% FCS and 1% Penicillin/Streptomycin (all from Hyclone) in a humidified incubator at 37 °C in 5% CO_2_. Cell line were authenticated by India (EBOT).

### 2.3. Cell Co-Culture

For co-culture experiments, each cell line was plated in a 48 well tissue culture-treated plate at a concentration of 0.1 × 10^6^ cells/well. Cells were kept in a humidified incubator at 37 °C in 5% CO_2_ for 4–5 h to allow cell adherence. Non-adherent cells were carefully removed and fresh complete medium was replaced prior to the addition of activated PBMCs. 

PBMCs were activated with 2 µg/mL of anti-CD3 (Clone OKT3) and 2 µg/mL of anti-CD28 (Clone CD28.2) antibodies (eBioscience, California, USA). To distinguish the effects of co-culturing PBMCs with breast cancer cells from PBMC activation, two controls were used; activated PBMCs cultured alone (negative control) or co-cultured with breast cancer cells from each cell line, in the absence of anti-PD-1 and anti-PD-L1 mAbs (control co-culture). Activated PBMCs and breast cancer cells were co-cultured at a ratio of 20:1. 

Cell lines were treated with 0.5 µg/mL of anti-PD-L1 mAb (atezolizumab; BioVision Inc., California USA) followed by the addition of activated PBMCs (2 × 10^6^ cells/well). In other wells, cell lines were co-cultured with activated PBMCs and treated with 2 µg/mL anti-PD-1 mAb (*pembrolizumab;* Keytruda® from Merck & Co., Inc., New Jersey, USA). For the combined blockade of PD-1 and PD-L1, activated PBMCs treated with anti-PD-1 mAb were co-cultured with cell lines treated with anti-PD-L1 mAb. Activated PBMCs were harvested at 24 h, 48 h and 72 h post mAb treatment for flow cytometric analyses.

### 2.4. Phenotypic Analyses by Flow Cytometry 

#### 2.4.1. Cell Surface Staining

Flow cytometric analyses were used to determine the cell surface expression of ICs including, PD-1, TIM-3 and LAG-3, on T cell subsets in the absence of mAb treatment or following the single and combined blockade of PD-L1 and PD-1. 

Cells were washed in phosphate-buffered saline (PBS), and re-suspended in 100 µL of staining buffer (PBS with 2% FCS and 0.1% sodium azide). Cells were blocked with a human IgG1 antibody (Sigma-Aldrich) for 10 min on ice. To gate out dead cells, Fixable Viability Dye eFluor 780 (FVD780; BioLegend, California, USA) was utilized. For surface staining, cells were stained with anti-CD4-Alexa Fluro 700 (Clone RPA-T4, BD Pharmingen, California, USA), anti-CD25-Brilliant Violet 650 (Clone M-A251, BioLegend), anti-PD-1-Phycoerythrin/Texas Red (PE-Dazzle^™^ 594) (Clone EH12.2H7, BioLegend), anti-TIM-3-Brilliant Violet 711 (Clone 7D3; BD Biosciences, California, USA), and anti-LAG-3-Brilliant Violet 421 (Clone T47-530; BD Biosciences) for 30 min at 4 °C in the dark.

#### 2.4.2. Intracellular Staining

For intracellular staining, cells were washed twice with staining buffer and fixed/permeabilized using fixation/permeabilization buffer (eBioscience) at 4 °C for 45 min. After two washes with permeabilization wash buffer (eBioscience), cells were blocked with mouse and rat serum (Sigma-Aldrich) for 10 min at 4 °C in the dark, then stained with anti-Helios-fluorescein isothiocyanate (FITC; Clone 22F6, Biolegend), anti-FoxP3-phycoerythrin cyanin 7 (PE/Cy7; Clone PCH101, eBioscience) and anti-CTLA-4-Peridinin Chlorophyll Protein Complex/e-Fluor™ 710 (PerCp-Fluor™ 710; Clone 14D3, eBioscience) antibodies for 30 min at 4 °C in the dark. Cells were washed twice with permeabilization buffer, and re-suspended in 300 µL of FACS staining buffer (eBioscience). Data were acquired by BD LSRFortessa X-20 flow cytometer (BD Biosciences) and analyzed by FlowJo v.10.0 software (Tree Star, Ashland, Covington, KY, USA).

The percentage of CD4^+^ T cells expressing a certain IC in activated PBMCs compared to control co-culture (breast cancer cell line + activated PBMCs) was used as a measure to determine upregulation or downregulation of IC expression. Similarly, we compared the percentage of CD4^+^ T cells expressing a certain IC in different co-culture conditions to that in control co-culture.

### 2.5. Statistical Analyses

All statistical analyses were performed using GraphPad Prism version 8.0 software (GraphPad Software, Inc., San Diego, CA, USA). We checked normality using Shapiro-Wilk normality test. Paired *t*-test was performed on samples that passed the normality test and nonparametric/Wilcoxon matched-pairs signed rank tests were performed for samples that did not show normal distribution. A *p*-value < 0.05 was considered to be statistically significant. The *P* values are represented as the following: *** *p* < 0.001, ** *p* < 0.01, * *p* < 0.05. Data are represented as the mean of percentage ± standard error of the mean (SEM).

## 3. Results

### 3.1. Kinetics of Immune Checkpoints, FoxP3 and Helios Expression in CD4^+^ T Cells 

We first investigated the kinetics of IC expression on CD4^+^ T cells at different time-points; 24 h, 48 h and 72 h post PBMC activation and anti-PD-1 and/or anti-PD-L1 mAb(s) treatment. Additionally, we examined the expression of FoxP3 and Helios which are well-known transcription factors for Tregs. FoxP3 is a marker of Tregs that positively regulates Treg differentiation/development and enhances their suppressive functions [25,26], while Helios is known for Treg stability and Treg suppressive functions [27,28]. 

**Figure 1:**
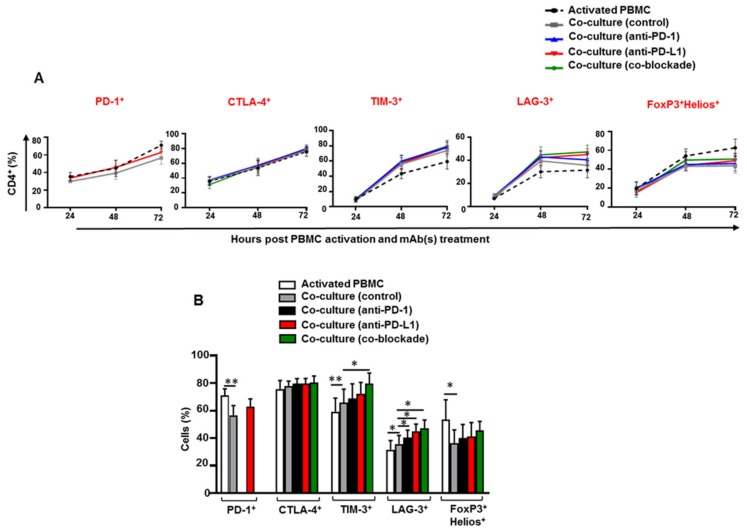
Kinetics of immune checkpoint expression on CD4^+^ T cells in co-culture with MDA-MB-231 cells. Activated peripheral blood mononuclear cells (PBMCs) co-cultured with MDA-MB-231 cells were treated with or without 2 µg/mL of anti-PD-1, 0.5 µg/mL of anti-PD-L1 or with both mAbs. Cells were stained for PD-1, CTLA-4, TIM-3, LAG-3, FoxP3 and Helios expression at 24 h, 48 h and 72 h, post PBMC activation and mAb treatment, and analyzed by flow cytometry. Line graphs show the kinetics of immune checkpoint and FoxP3/Helios expression in CD4^+^ T cells (**A**). Bar plot shows the percentage of CD4^+^ T cells expressing different markers for each experimental group at 72 h (**B**). Data represent the mean + SEM of four independent experiments.

We found that the percentage of CD4^+^PD-1^+^, CD4^+^CTLA-4^+^ and CD4^+^TIM-3^+^ T cells increased over the 3 days period following PBMC activation (Figure 1A). The percentage of CD4^+^LAG-3^+^ and CD4^+^FoxP3^+^Helios^+^ T cells increased at 48 h and sustained until 72 h (Figure 1A). The expression levels of TIM-3, LAG-3 and FoxP3/Helios differed between activated PBMCs and control co-culture or between control co-culture and mAb-treated co-cultures were only seen at the 72-h time-point (Figure 1B). Hence, this time-point was selected for all of subsequent analyses.

### 3.2. Breast Cancer Cells Upregulate IC Expression on CD4^+^CD25^−^ T Cell Subset 

**Figure 2:**
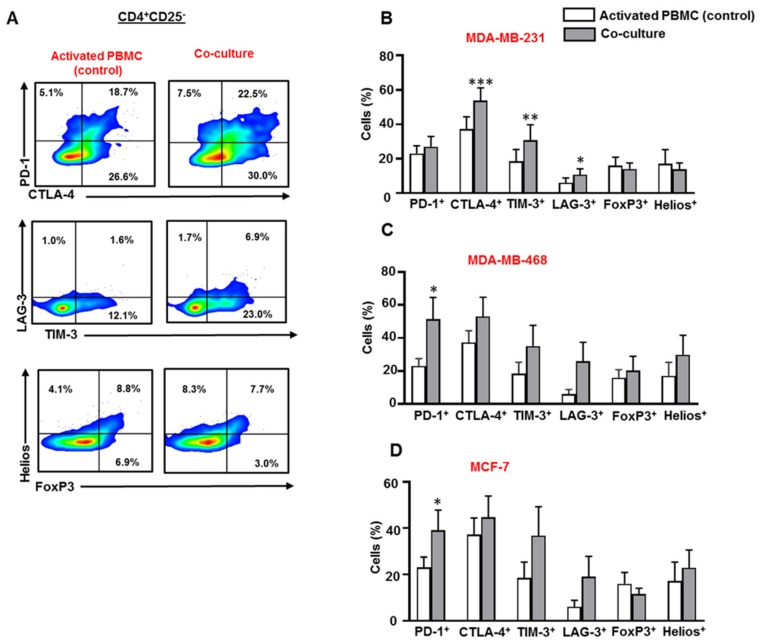
Immune checkpoint and Treg-related marker expression on CD4^+^CD25^−^ T cells in co-culture with breast cancer cells. Activated PBMCs were co-cultured with MDA-MB-231, MDA-MB-468, and MCF-7 cells for 72 h. Cells were then stained for immune checkpoints and FoxP3/Helios expression, and analyzed by flow cytometry. Representative flow cytometric plots show PD-1, CTLA-4, TIM-3, LAG-3, FoxP3 and Helios expression in CD4^+^CD25^−^ T cells from activated PBMC and MDA-MB-231 co-culture (**A**). Bar plots show the differences in IC, FoxP3 and Helios expression in CD4^+^CD25^−^ T cells in the presence or absence of MDA-MB-231 (**B**), MDA-MB-468 (**C**) and MCF-7 (**D**) cells. Data represent the mean + SEM of four independent experiments.

Next, we examined the expression of PD-1, CTLA-4, LAG-3, TIM-3, FoxP3, and Helios in CD4^+^CD25^−^ T cell subset in the presence or absence of breast cancer cells (Figure 2A). Our data showed that the combination of upregulated ICs differs from one cell line to another. We found that MDA-MB-231 cells upregulated CTLA-4, TIM-3 and LAG-3 expression in CD4^+^CD25^−^ T cells (Figure 2B). However, MDA-MB-468 cells upregulated PD-1 expression and showed a trend towards upregulated levels of CTLA-4, TIM-3 and LAG-3 expression in CD4^+^CD25^−^ T cells (Figure 2C). MCF-7 cells also upregulated the expression of PD-1 and showed a trend towards upregulated levels of TIM-3 and LAG-3 on CD4^+^CD25^−^ T cells (Figure 2D). The expression of FoxP3 and Helios in CD4^+^CD25^−^ T cells was relatively similar (Figure 2B–D). 

### 3.3. Breast Cancer Cells Upregulate IC Expression on Activated CD4^+^CD25^+^ T Cell Subset 

**Figure 3:**
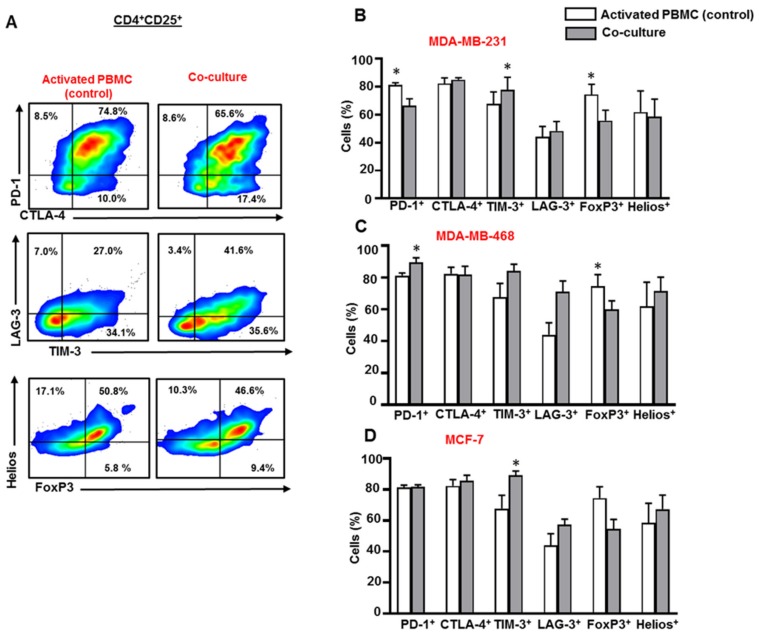
Immune checkpoint and Treg-related marker expression on CD4^+^CD25^+^ T cells in co-culture with breast cancer cells. Activated PBMCs were co-cultured with MDA-MB-231, MDA-MB-468, and MCF-7 cells for 72 h. Cells were then stained for immune checkpoints and FoxP3/Helios expression, and analyzed by flow cytometry. Representative flow cytometric plots show PD-1, CTLA-4, TIM-3, LAG-3, FoxP3 and Helios expression in CD4^+^CD25^+^ T cells from activated PBMC and MDA-MB-231 co-culture (**A**). Bar plots show the differences in IC, FoxP3 and Helios expression in CD4^+^CD25^+^ T cells in the presence or absence of MDA-MB-231 (**B**), MDA-MB-468 (**C**) and MCF-7 (**D**) cells. Data represent the mean + SEM of four independent experiments.

Next, we investigated IC, FoxP3 and Helios expression in activated CD4^+^CD25^+^ T cells, in the presence or absence of breast cancer cells (Figure 3A). MDA-MB-231 cells downregulated PD-1 expression on CD4^+^CD25^+^ T cells (Figure 3B), but upregulated the expression of TIM-3 on CD4^+^CD25^+^ T cells (Figure 3B). Unlike MDA-MB-231, MDA-MB-468 cells upregulated the expression of PD-1 and showed a trend towards upregulated levels of TIM-3 and LAG-3 on CD4^+^CD25^+^ T cells (Figure 3C). Both MDA-MB-231 (Figure 3B) and MDA-MB-468 (Figure 3C) cells downregulated the expression of FoxP3 in CD4^+^CD25^+^ T cells. MCF-7 cells upregulated the expression of TIM-3 on CD4^+^CD25^+^ T cells (Figure 3D). 

### 3.4. The Co-Blockade of PD-1 and PD-L1 Downregulates CTLA-4 Expression in CD4^+^CD25^−^ T Cells in the Presence of TNBC Cells

**Figure 4:**
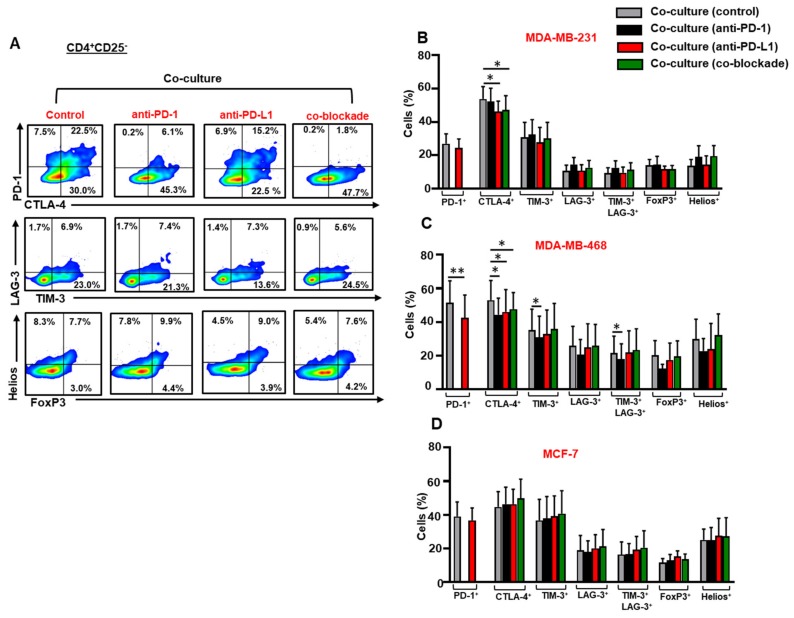
Effect of blocking PD-1 and PD-L1 on immune checkpoint and Treg-related marker expression in CD4^+^CD25^−^ T cells. Activated PBMCs co-cultured with breast cancer cells were treated or untreated with 2 µg/mL of anti-PD-1, 0.5 µg/mL of anti-PD-L1 or with both mAbs. At 72 h post mAb treatment, cells were stained for immune checkpoints and FoxP3/Helios expression, and analyzed by flow cytometry. Representative flow cytometric plots show PD-1, CTLA-4, TIM-3, LAG-3, FoxP3 and Helios expression in CD4^+^CD25^−^ T cells from MDA-MB-231 co-culture, in the presence or absence of mAb(s) (**A**). Bar plots show the differences in IC, FoxP3 and Helios expression in CD4^+^CD25^−^ T cells in the presence or absence of MDA-MB-231 (**B**), MDA-MB-468 (**C**) and MCF-7 (**D**) cells. Data represent the mean + SEM of four independent experiments.

We investigated the effect of anti-PD-1, anti-PD-L1 or both mAbs on the expression of ICs, FoxP3 and Helios in CD4^+^CD25^−^ T cells in the presence of breast cancer cells (Figure 4A). The blockade of PD-L1 alone or in combination with PD-1 downregulated the expression of CTLA-4 in CD4^+^CD25^−^ T cells in the presence MDA-MB-231 (Figure 4B) and MDA-MB-468 (Figure 4C) cells. Anti-PD-L1 mAb downregulated the expression of PD-1 on CD4^+^CD25^−^ T cells in the presence of MDA-MB-468 cells (Figure 4C). Anti-PD-1 mAb alone downregulated the expression of TIM-3 and TIM-3/LAG-3 co-expression on CD4^+^CD25^−^ T cells in the presence of MDA-MB-468 cells (Figure 4C). The single or co-blockade of PD-1 and PD-L1 did not have any effects on the expression of ICs or Treg-related markers in CD4^+^CD25^−^ T cells in the presence of MCF-7 cells (Figure 4D). 

### 3.5. The Co-Blockade of PD-1 and PD-L1 Further Upregulates TIM-3/LAG-3 co-expression on CD4^+^CD25^+^ T Cells in the Presence of Breast Cancer Cells

**Figure 5:**
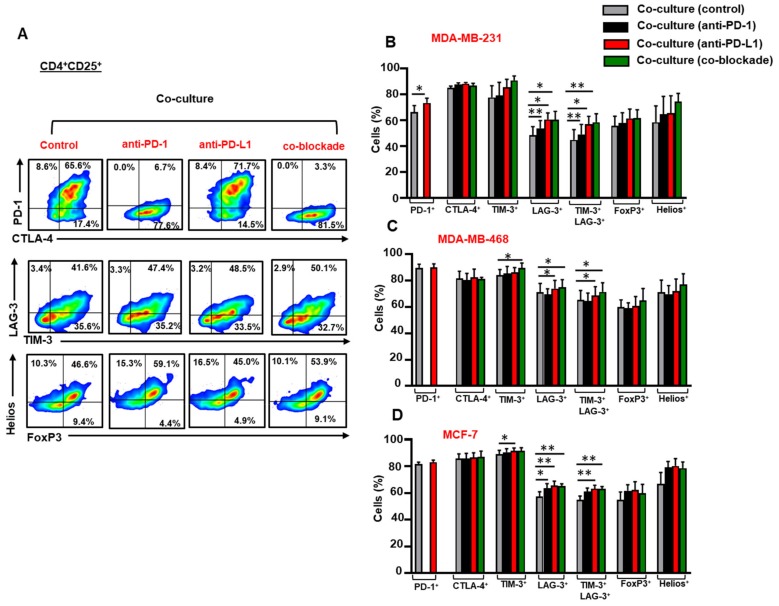
Effect of blocking PD-1 and PD-L1 on immune checkpoint and Treg-related marker expression in CD4^+^CD25^+^ T cells. Activated PBMCs co-cultured with breast cancer cells were treated or untreated with 2 µg/mL of anti-PD-1, 0.5 µg/mL of anti-PD-L1 or with both mAbs. At 72 h post mAb treatment, cells were stained for immune checkpoints and FoxP3/Helios expression, and analyzed by flow cytometry. Representative flow cytometric plots show PD-1, CTLA-4, TIM-3, LAG-3, FoxP3 and Helios expression in CD4^+^CD25^+^ T cells from MDA-MB-231 co-culture, in the presence or absence of mAb(s) (**A**). Bar plots show the differences in IC, FoxP3 and Helios expression in CD4^+^CD25^+^ T cells in the presence or absence of MDA-MB-231 (**B**), MDA-MB-468 (**C**) and MCF-7 (**D**) cells. Data represent the mean + SEM of four independent experiments.

Next, we examined the effect of anti-PD-1, anti-PD-L1 or both mAbs on the expression of ICs, FoxP3 and Helios in CD4^+^CD25^+^ T cells in the presence of breast cancer cells (Figure 5A). Anti-PD-L1 further upregulated the expression of PD-1 on CD4^+^CD25^+^ T cells in the presence of MDA-MB-231 cells (Figure 5B). The single blockade of PD-L1 or in combination with PD-1 further enhanced the expression of LAG-3 and the co-expression of TIM-3 and LAG-3 on CD4^+^CD25^+^ T cells in the presence of MDA-MB-231 (Figure 5B), MDA-MB-468 cells (Figure 5C) and MCF-7 (Figure 5D) cells. 

Anti-PD-1 mAb alone, on the other hand, did not show any effects on IC expression on CD4^+^CD25^+^ T cells in the presence of MDA-MB-468 cells (Figure 5C), in contrast to MDA-MB-231 (Figure 5B) and MCF-7 (Figure 5D) cells. 

Anti-PD-1, anti-PD-L1 or both mAbs further upregulated the expression of LAG-3 on CD4^+^CD25^+^ T cells in the presence of MCF-7 cells (Figure 5D). Anti-PD-L1 mAb and the co-blockade further enhanced the co-expression of TIM-3 and LAG-3 on CD4^+^CD25^+^ T cells in the presence of MCF-7 cells (Figure 5D). Our data demonstrated that the co-blockade of PD-1 and PD-L1 has no effect on CTLA-4 expression on CD4^+^CD25^+^ T cells in the presence of TNBC and non-TNBC cells. 

### 3.6. Breast Cancer Cells Upregulate TIM-3 and LAG-3 Co-Expression on Tregs which is Further Upregulated Following the Co-Blockade of PD-1 and PD-L1 in the Presence of TNBC Cells

**Figure 6:**
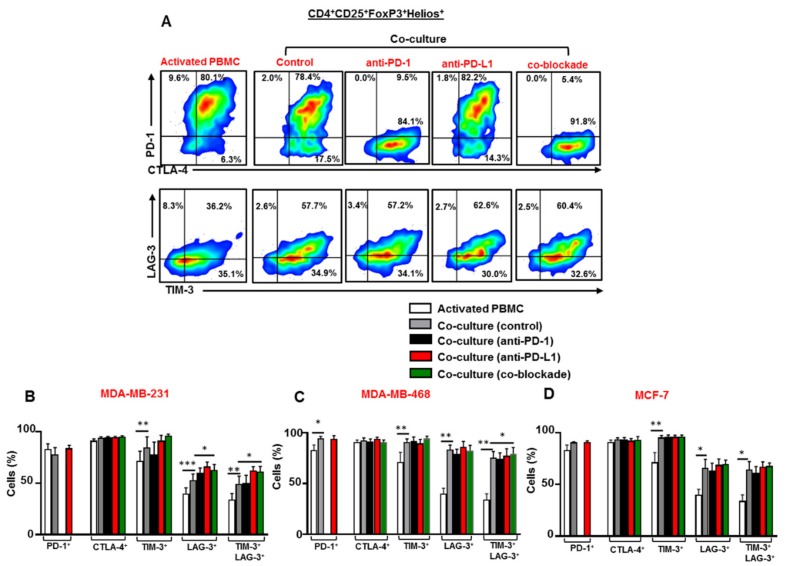
Effect of blocking PD-1 and PD-L1 on immune checkpoint expression in CD4^+^CD25^+^FoxP3^+^Helios^+^ Tregs. Activated PBMCs co-cultured with breast cancer cells were treated or untreated with 2 µg/mL of anti-PD-1, 0.5 µg/mL of anti-PD-L1 or with both mAbs. At 72 h post-mAb treatment cells were stained for immune checkpoints and FoxP3/Helios expression, and analyzed by flow cytometry. Representative flow cytometric plots show the expression of PD-1, CTLA-4, TIM-3 and LAG-3 in Tregs from activated PBMC and MDA-MB-231 co-culture treated or untreated with mAb(s) (**A**). Bar plots show the differences in IC expression on Tregs in the presence or absence of MDA-MB-231 (**B**), MDA-MB-468 (**C**) and MCF-7 (**D**) cells. Data represent the mean + SEM of four independent experiments.

We investigated the effect of breast cancer cells on the expression level of ICs on Tregs (defined as CD4^+^CD25^+^FoxP3^+^Helios^+^), in addition to the effect of anti-PD-1, anti-PD-L1 or both mAbs on the expression of ICs on Tregs in the presence of breast cancer cells (Figure 6A). Our data showed elevated levels of TIM-3 and LAG-3 co-expression on Tregs in the presence of MDA-MB-231 (Figure 6B), MDA-MB-468 (Figure 6C) and MCF-7 (Figure 6D) cells. This co-expression of TIM-3 and LAG-3 was further upregulated on Tregs following the co-blockade of PD-1 and PD-L1 in the presence of TNBC cells. However, the co-blockade of PD-1 and PD-L1 in the presence of MCF-7 cells had no effects on co-the expression of TIM-3 and LAG-3 on Tregs. 

Collectively, we found that breast cancer cells upregulated the expression of PD-1, CTLA-4, TIM-3 and LAG-3 in the different subsets of CD4^+^ T cells. The combinations of ICs upregulated by breast cancer cells differed from one cell line to another, and across the different CD4^+^ T cell subsets. More importantly, we showed that TNBC cells further upregulated the co-expression TIM-3 and LAG-3 on CD4^+^CD25^+^ T cells and CD4^+^CD25^+^FoxP3^+^Helios^+^ Tregs following the co-blockade of PD-1 and PD-L1. This finding suggests the emergence of compensatory inhibitory mechanisms, which could lead to the development of TNBC resistance to the co-blockade of PD-1 and PD-L1. 

## 4. Discussion

Within the TME, cancer cells exert suppressive activities on T effector cells (Teffs) and alter their immune phenotype by secreting soluble molecules or inducing the expression of co-inhibitory receptors such as ICs [29,30]. Immunotherapy in the form of ICIs have been developed, with some currently in clinical trials and others have been approved for the treatment of different cancers. However, tumor cells can acquire additional inhibitory pathways to counteract the activity of ICIs and mediate immune evasion, for example by inducing the expression of other ICs or IC ligands within TME [31]. This in turn leads to the development of acquired resistance to immunotherapy [32]. Therefore, it is crucial to advance our understanding on the role of ICs in the TME and the mechanisms that facilitate resistance development.

The expression of key ICs, such as PD-1, CTLA-4, TIM-3 and LAG-3, on CD4^+^ or CD8^+^ T cells generates inhibitory signals which suppress the proliferation and activation of Teffs or causes exhaustion [23,33,34]. Moreover, increased expression or co-expression of inhibitory ICs, such as PD-1, TIM-3 and LAG-3, has been considered to be an indication of Teff exhaustion [35]; however, further studies are required to investigate the expression of transcription factors associated with T cell exhaustion, such as NFAT, T-bet, EOMES, FOXO1 and FOXP1 [36] and TOX [37]. Conversely, upregulated levels of ICs on Tregs enhance their suppressive activity, survival and expansion [38,39,40].

We found that TNBC (MDA-MB-231 and MDA-MB-468) and non-TNBC cells (MCF-7) upregulate the expression of PD-1, CTLA-4, TIM-3 and/or LAG-3 in CD4^+^CD25^−^ T cells (comprising of non-Tregs and non-activated T cells). Breast cancer cells also upregulated the expression of PD-1, TIM-3 and/or LAG-3, but not CTLA-4, in CD4^+^CD25^+^ T cells (comprising of activated T cells and Tregs). PD-1, TIM-3 and LAG-3 are mainly detected on T cells upon activation [23]. CTLA-4, on the other hand, is constitutively expressed by Tregs, and induced in other T cells upon activation [23]. This could explain why the level of CTLA-4 expression in CD4^+^CD25^+^ T cells did not differ in the presence or absence of breast cancer cells. The levels of LAG-3 expression on CD4^+^CD25^+^ T cells were approximately 20% higher in the presence of MDA-MB-468 cells, compared to those found in MDA-MB-231 co-culture. These results suggest a possible negative correlation between LAG-3 expression on activated T cells and PD-L1 expression on breast cancer cells.

The fact that MDA-MB-231 and MDA-MB-468 cells express PD-L1 at different levels could be responsible for the differential effects on PD-1 expression and TIM-3/LAG-3 co-expression in CD4^+^CD25^−^ T cells. It seems that blocking PD-L1 in TNBC cells that originally express lower level of PD-L1 (MDA-MB-468) is more sufficient in reducing PD-1 and TIM-3/LAG-3 levels in non-activated CD4^+^CD25^−^ T cells than blocking PD-L1 in MDA-MB-231 cells. FoxP3 expression can be induced transiently upon activation of T cells [41,42]; however, its expression is sustained in canonical Tregs. We found that FoxP3 expression in CD4^+^CD25^+^ T cells was lower in the presence of TNBC cells than that in the control activated PBMC. This finding suggests that TNBC cells may release mediators, for example, reactive oxygen species (ROS) to trigger T cell apoptosis, and that FoxP3^+^ Tregs are more susceptible to cell death than Teffs [43]; hence FoxP3 levels were reduced. 

The blockade of particular ICs usually results in the development of compensatory inhibitory mechanisms such as the upregulation of other ICs by which immunosuppressive cells promote tumorigenesis [44,45,46]. In accordance with this, we found that the single blockade of PD-L1 or in combination with PD-1 did not have any beneficial effects on activated CD4^+^CD25^+^ T cells but, in fact, further increased the expression of alternate ICs, such as TIM-3 and LAG-3 (new emerging therapeutic targets for cancer). The inhibition of PD-1 or PD-L1 has been associated with the upregulation of TIM-3 on T cells in mouse lung cancer model [47] and head and neck cancer model [45], respectively. In another study, the blockade of PD-1 resulted in the up-regulation of LAG-3 on CD8^+^ T cells in mouse ovarian cancer model [44]. Together, these data suggest that upregulated levels of TIM-3 and LAG-3 on T cells are potential compensatory mechanisms, which may lead to the development of acquired resistance to anti-PD-1 and PD-L1 mAbs (as reviewed in [31,32]). The co-blockade of TIM-3 and PD-L1 [45] or LAG-3 and PD-1 [44] enhanced the therapeutic efficacy, and increased the ratio of cytotoxic T cells: Tregs in the tumor compared to targeting PD-L1 or PD-1 alone. The therapeutic efficacy of targeting TIM-3 and LAG-3 is currently under clinical investigation in a range of cancer patients [23]. In addition, the combined inhibition of TIM-3 or LAG-3 with anti-PD-1/PD-L1 mAb could provide better therapeutic means for enhancing Teff activity, targeting Treg function and overcoming resistance, thereby maximizing the efficacy of current cancer therapies. 

In the presence of MDA-MB-231 cells, the blockade of PD-L1 further enhanced the expression of PD-1 on CD4^+^CD25^+^ T cells, but had no effect on PD-1 expression in the presence MDA-MB-468 cells. Once again, this could be attributed to the differential expression of PD-L1 in each cell line, with MDA-MB-231 being more responsive to anti-PD-L1 than MDA-MB-468 cells, suggesting that the blockade of the ligand upregulates the expression of the receptor on CD4^+^CD25^+^ T cells. 

FoxP3^+^Helios^+^ Tregs are considered to be more immunosuppressive and highly activated than FoxP3^+^Helios^−^ Tregs, characterized by producing high levels of IL-10 [48]. Increased numbers of FoxP3^+^Helios^+^ Tregs expressing upregulated levels of ICs have been associated with poor disease prognosis in cancer patients, for example, those with colorectal cancer [49]. We found that the co-expression of TIM-3/LAG-3 was upregulated in FoxP3^+^Helios^+^ Tregs in the presence of breast cancer cells. Consistent with this, it has been reported that the emergence of TIM-3^+^ Tregs compensates for PD-L1 blockade, and causes resistance to therapy, tumor relapse and progression in head and neck mouse cancer model [45]. 

Unlike the blockade of PD-L1, anti-PD-1 alone did not further upregulate TIM-3 and LAG-3 co-expression on CD4^+^CD25^+^FoxP3^+^Helios^+^ Tregs in the presence of breast cancer cells. This implies that targeting PD-1 in breast cancer could be a better therapeutic approach than targeting the ligand as it is less likely to result in the upregulation of alternate ICs.

## 5. Conclusions

Our findings indicate that TNBC cells upregulate the expression of inhibitory ICs in different CD4^+^ T cell subsets including Tregs. We also found that TNBC cells and the co-blockade of PD-1 and PD-L1 further upregulate the co-expression of TIM-3 and LAG-3 on CD4^+^CD25^+^ T cells, including Tregs. This suggests the emergence of compensatory inhibitory mechanisms, which could lead to the development of TNBC resistance against PD-1/PD-L1 inhibition.
